# Supplementary material for: Cytotoxicity of the effector protein BteA was attenuated in Bordetella pertussis by insertion of an alanine residue
Source: PLoS Pathog. 2020 Aug 10;16(8):e1008512. doi: 10.1371/journal.ppat.1008512 (PMC7446853; doi:10.1371/journal.ppat.1008512)
Supplement: S1 Fig — Alignment of amino acid residues 488–519 of BteA effector protein of representative Bordetella species. Bp BteA of B. pertussis complex II lineage I: 18323 (Bp18323, WP_014905434.1), complex II lineage II: Tohama I (BpTohama I, WP_010929841.1), B1917 (BpB1917, WP_010929841.1), B1920 (BpB1920, WP_010929841.1), Bb BteA of B. bronchiseptica complex I: RB50 (BbRB50, WP_003814629.1), 253 (Bb253, WP_003814629.1), complex IV: D445 (BbD445, WP_004567631.1), Bbr77 (BbBbr77, WP_003820735.1), and Bpp BteA of B. parapertussis ovine: Bpp5 (BppBpp5, WP_003814629.1) and human: 12822 (Bpp12822, WP_010929242.1) is shown. Presence of alanine at position 503 is highlighted in red. (PDF) [file ppat.1008512.s001.pdf]

	488	489	490	491	492	493	494	495	496	497	498	499	500	501	502	503	504	505	506	507	508	509	510	511	512	513	514	515	516	517	518	519
Bp 18323	Y	A	D	A	H	G	L	T	A	S	V	T	G	S	A	**A**	N	V	G	L	G	A	T	A	E	G	K	L	V	A	S	P
Bp Tohama I	Y	A	D	A	H	G	L	T	A	S	V	T	G	S	A	**A**	N	V	G	L	G	A	T	A	E	G	K	L	V	A	S	P
Bp B1917	Y	A	D	A	H	G	L	T	A	S	V	T	G	S	A	**A**	N	V	G	L	G	A	T	A	E	G	K	L	V	A	S	P
Bp B1920	Y	A	D	A	H	G	L	T	A	S	V	T	G	S	A	**A**	N	V	G	L	G	A	T	A	E	G	K	L	V	A	S	P
Bb RB50	Y	A	D	A	H	G	L	T	A	S	V	T	G	S	A	-	N	V	G	L	G	A	T	A	E	G	K	L	V	A	S	P
Bb 253	Y	A	D	A	H	G	L	T	A	S	V	T	G	S	A	-	N	V	G	L	G	A	T	A	E	G	K	L	V	A	S	P
Bb D445	Y	A	E	A	H	G	L	T	A	S	V	T	G	S	A	-	N	V	G	L	G	A	T	A	E	G	K	L	V	A	S	P
Bb Bbr77	Y	A	E	A	H	G	L	T	A	S	V	T	G	S	A	-	N	V	G	L	G	A	T	A	E	G	K	L	V	A	S	P
Bpp Bpp5	Y	A	D	A	H	G	L	T	A	S	V	T	G	S	A	-	N	V	G	L	G	A	T	A	E	G	K	L	V	A	S	P
Bpp 12822	Y	A	E	A	H	G	L	T	A	S	V	T	G	S	A	-	N	V	G	L	G	A	T	A	E	G	K	L	V	A	S	P

S1 Figure. All isolates of *B. pertussis* carry alanine at position 503 in their *Bp* BteA protein unlike isolates of *B. bronchiseptica* and *B. parapertussis* species.

Alignment of amino acid residues 488 – 519 of BteA effector protein of representative *Bordetella* species. *Bp* BteA of *B. pertussis* complex II lineage I: 18323 (*Bp*18323, WP_014905434.1), complex II lineage II: Tohama I (*Bp*Tohama I, WP_010929841.1), B1917 (*Bp*B1917, WP_010929841.1), B1920 (*Bp*B1920, WP_010929841.1), *Bb* BteA of *B. bronchiseptica* complex I: RB50 (*Bb*RB50, WP_003814629.1), 253 (*Bb*253, WP_003814629.1), complex IV: D445 (*Bb*D445, WP_004567631.1), Bbr77 (*Bb*Bbr77, WP_003820735.1), and *Bpp* BteA of *B. parapertussis* ovine: Bpp5 (*Bpp*Bpp5, WP_003814629.1) and human: 12822 (*Bpp*12822, WP_010929242.1) is shown. Presence of alanine at position 503 is highlighted in red.
