# Supplementary material for: Cytotoxicity of the effector protein BteA was attenuated in Bordetella pertussis by insertion of an alanine residue
Source: PLoS Pathog. 2020 Aug 10;16(8):e1008512. doi: 10.1371/journal.ppat.1008512 (PMC7446853; doi:10.1371/journal.ppat.1008512)
Supplement: S1 Table — Bacterial strain name, genotype description and reference are indicated. (PDF) [file ppat.1008512.s002.pdf]

S1 Table. List of bacterial strains used in this study. Bacterial strain name, genotype description and reference are indicated.

**Strain**	**Genotype and relevant description**	**Reference**
Escherichia coli strains		
XL1-Blue	*rec*A1 *end*A1 *gyr*A96 *thi*-1 *hsd*R17 *sup*E44 *rel*A1 *lac* F´ *pro*AB l*ac*IqZΔM15 Tn10 Tet^r	Stratagene
SM10 λpir	*thi thr leu tonA lacY supE recA*::RP4-2-Tc::Mu Km λ*pir*	[1, 2]
Bordetella bronchiseptica strains		
WT	*Bb*D445 WT; wild type *Bordetella bronchiseptica* MO211 (B2495); complex IV human isolate; ST-17	[3]
Δ*bscN*	*Bb*D445 Δ*bscN; Bb*D445 strain derivative with *bscN* in-frame deletion of codons R2-E443	this study
Δ*bteA*	*Bb*D445 Δ*bteA; Bb*D445 strain derivative with *bteA* in-frame deletion of codons L2-A657	this study
*bteA*insA503	*Bb*D445 *bteA*insA503; *Bb*D445 strain derivative with *bteA* in-frame insertion of codon A at position 503	this study
Δ*bteA* / *Bb*D445 *bteA*	*Bb*D445 Δ*bteA* strain derivative harboring pBBRI-encoded *bteA* allele of *Bb*D445	this study
ΔbteA / *Bb*RB50 *bteA*	*Bb*D445 Δ*bteA* strain derivative harboring pBBRI-encoded *bteA* allele of *Bb*RB50	this study
ΔbteA / *Bp bteA*	*Bb*D445 Δ*bteA* strain derivative harboring pBBRI-encoded *bteA* allele of *Bp*B1917	this study
ΔbteA / *Bp bteA*A449T	*Bb*D445 Δ*bteA* strain derivative harboring pBBRI-encoded *Bp*B1917 *bteA*A449T mutant allele	this study
ΔbteA / *Bp bteA*S460G	*Bb*D445 Δ*bteA* strain derivative harboring pBBRI-encoded *Bp*B1917 *bteA*S460G mutant allele	this study
ΔbteA / *Bp bteA*A465G	*Bb*D445 Δ*bteA* strain derivative harboring pBBRI-encoded *Bp*B1917 *bteA*A465G mutant allele	this study
ΔbteA / *Bp bteA*ΔA503	*Bb*D445 Δ*bteA* strain derivative harboring pBBRI-encoded *Bp*B1917 *bteA*ΔA503 mutant allele	this study
ΔbteA / *Bp bteA*insGVE	*Bb*D445 Δ*bteA* strain derivative harboring pBBRI-encoded *Bp*B1917 *bteA*insGVE610 mutant allele	this study
*Bb*RB50 WT	*Bb*RB50 WT; wild type *Bordetella bronchiseptica* RB50 (B1976); complex I rabbit isolate; ST-12	[3, 4]

S1 Table. Continued

Bordetella pertussis strains		
Bp WT	*Bp*B1917 WT; wild type *Bordetella pertussis* 1917*; fim2-1, fim3-2, ptxP3, ptxA1, ptxB2, ptxC2, ptxD1, ptxE1, prn2*	[5, 6]
Bp Δ*bscN*	*Bp*B1917 Δ*bscN; Bp*B1917 strain derivative with *bscN* in-frame deletion of codons R2-E443	this study
Bp Δ*bteA*	*Bp*B1917 Δ*bteA; Bp*B1917 strain derivative with *bteA* in-frame deletion of codons L2-A656	this study
*Bp bteA*ΔA503	*Bp*B1917 *bteA* ΔA503; *Bp*B1917 strain derivative with *bteA* in-frame deletion of codon A503	this study
Bp Δ*btrA*	*Bp*B1917 Δ*btrA; Bp*B1917 strain derivative with *btrA* in-frame deletion of codons C7-L223	this study

References

1. Simon R, Priefer U, Pühler A. A Broad Host Range Mobilization System for In Vivo Genetic Engineering: Transposon Mutagenesis in Gram Negative Bacteria. Bio/Technology. 1983;1:784. doi: 10.1038/nbt1183-784.
2. Skopova K, Tomalova B, Kanchev I, Rossmann P, Svedova M, Adkins I, et al. Cyclic AMP-Elevating Capacity of Adenylate Cyclase Toxin-Hemolysin Is Sufficient for Lung Infection but Not for Full Virulence of Bordetella pertussis. Infect Immun. 2017;85(6). doi: 10.1128/IAI.00937-16. PubMed PMID: 28396322; PubMed Central PMCID: PMCPMC5442630.
3. Diavatopoulos DA, Cummings CA, Schouls LM, Brinig MM, Relman DA, Mooi FR. Bordetella pertussis, the causative agent of whooping cough, evolved from a distinct, human-associated lineage of B. bronchiseptica. Plos Pathog. 2005;1(4):e45. doi: 10.1371/journal.ppat.0010045. PubMed PMID: 16389302; PubMed Central PMCID: PMC1323478.
4. Cotter PA, Miller JF. BvgAS-mediated signal transduction: analysis of phase-locked regulatory mutants of Bordetella bronchiseptica in a rabbit model. Infect Immun. 1994;62(8):3381-90. PubMed PMID: 8039908; PubMed Central PMCID: PMCPMC302969.
5. Bart MJ, Zeddeman A, van der Heide HG, Heuvelman K, van Gent M, Mooi FR. Complete Genome Sequences of Bordetella pertussis Isolates B1917 and B1920, Representing Two Predominant Global Lineages. Genome Announc. 2014;2(6). doi: 10.1128/genomeA.01301-14. PubMed PMID: 25540342; PubMed Central PMCID: PMC4276820.
6. Bart MJ, Harris SR, Advani A, Arakawa Y, Bottero D, Bouchez V, et al. Global population structure and evolution of Bordetella pertussis and their relationship with vaccination. MBio. 2014;5(2):e01074. doi: 10.1128/mBio.01074-14. PubMed PMID: 24757216; PubMed Central PMCID: PMC3994516.
