# Supplementary material for: Cytotoxicity of the effector protein BteA was attenuated in Bordetella pertussis by insertion of an alanine residue
Source: PLoS Pathog. 2020 Aug 10;16(8):e1008512. doi: 10.1371/journal.ppat.1008512 (PMC7446853; doi:10.1371/journal.ppat.1008512)
Supplement: S2 Table — Plasmid name, description and reference are provided. (PDF) [file ppat.1008512.s003.pdf]

S2 Table. List of plasmids used in this study. Plasmid name, description and reference are provided.

**Plasmid**	**Description**	**Reference**
pSS4245	Allelic exchange vector for *Bordetella spp.*, contains *ptx* promoter, *I-SceI*, *oriV*, *AmpR, StrR, KmR, BleR, TetR* and an *I-SceI* cleavage site for counterselection	[1, 2]
pSS4245 *Bb*D445 *ΔbscN*	pSS4245 vector containing homology regions h1 (681 bp, 3930866-3931546) and h2 (661 bp, 3928879-3929539) flanking in-frame deletion of codons R2-E443 in the *bscN* gene of *Bb*D445	this study
pSS4245 *Bb*D445 *ΔbteA*	pSS4245 vector containing homology regions h1 (713 bp, 3179951-3180663) and h2 (622 bp, 3182632-3183253) flanking in-frame deletion of codons L2-A657 in the *bteA* gene of *Bb*D445	this study
pSS4245 *Bb*D445 *bteA* insA503	pSS4245 vector containing homology regions h1 (659 bp, 3181508-3182166) and h2 (632 bp, 3182167-3182798) flanking in-frame insertion of codon A at position 503 in the *bteA* gene of *Bb*D445	this study
pSS4245 *Bp*B1917 *ΔbscN*	pSS4245 vector containing homology regions h1 (681 bp, 2285302-2285982) and h2 (661 bp, 2283315-2283975) flanking in-frame deletion of codons R2-E443 in the *bscN* gene of *Bp*B1917	this study
pSS4245 BpB1917 *ΔbteA*	pSS4245 vector containing homology regions h1 (712 bp, 3764220-3764931) and h2 (622 bp, 3761633-3762254) flanking in-frame deletion of codons L2-A656 in the *bteA* gene of *Bp*B1917	this study
pSS4245 *Bp*B1917 *bteA*ΔA503	pSS4245 vector containing homology regions h1 (659 bp, 3762717-3763375) and h2 (626 bp, 3762088-3762713) flanking in-frame deletion of the codon A503 in the *bteA* gene of *Bp*B1917	this study
pSS4245 *Bp*B1917 *ΔbtrA*	pSS4245 vector containing homology regions h1 (518 bp, 2274858-2275375) and h2 (466 bp, 2276027-2276492) flanking in-frame deletion of codons C7-L223 in the *btrA* gene of *Bp*B1917	this study
pBBRI MCS	*lacPOZ' mob*$^{+}$, broad-host cloning vector, Cm^{R}	[3, 4]
pBBRI *Bb*D445 *bteA*	pBBRI vector with promotor and coding sequence of the *bteA* allele of *Bb*D445, region: 3180419-3182634	this study
pBBRI *Bb*RB50 *bteA*	pBBRI vector with promotor and coding sequence of the *bteA* allele of *Bb*RB50, region: 4501813-4504030	this study
pBBRI *Bp bteA*	pBBRI vector with promotor and coding sequence of the *bteA* allele of *Bp*B1917, region: 3762252-3764463	this study
pBBRI *Bp bteA*A449T	pBBRI *Bp bteA* vector with A449T substitution in the *bteA* allele	this study
pBBRI *Bp bteA*S460G	pBBRI *Bp bteA* vector with S460G substitution in the *bteA* allele	this study
pBBRI *Bp bteA*A465G	pBBRI *Bp bteA* vector with A465G substitution in the *bteA* allele	this study

pBBRI *Bp bteA*ΔA503	pBBRI *Bp bteA* vector with ΔA503 codon deletion in the *bteA* allele	this study
pBBRI *Bp bteA*insGVE	pBBRI *Bp bteA* vector with insertion of tripeptide GVE at position 610 in the *bteA* allele	this study
pYC2-CT	Expression vector for *S. cerevisiae*, *GAL1* promoter, *URA3* marker, AmpR, *CEN6*/ARS	Invitrogen
pYC2-CT *Bb*RB50 *bteA-GFP*	pYC2-CT vector containing the *bteA* allele of *Bb*RB50 fused with *GFP* on its C-terminus, gene is under the control of the *GAL1* promoter	this study
pYC2-CT *Bb*RB50 *bteA*insA503-*GFP*	pYC2-CT *Bb*RB50 *bteA-GFP* vector with insertion of codon A at position 503 within the *bteA* allele, gene is under the control of the *GAL1* promoter	this study
pYC2-CT *Bp bteA-GFP*	pYC2-CT vector containing the *bteA* allele of *Bp*B1917 fused with *GFP* on its C-terminus, gene is under the control of the *GAL1* promoter	this study
pYC2-CT *Bp bteA*ΔA503 GFP	pYC2-CT *Bp bteA-GFP* vector with deletion of codon A at position 503 within the *bteA* allele, gene is under the control of the *GAL1* promoter	this study

References

1. Inatsuka CS, Xu Q, Vujkovic-Cvijin I, Wong S, Stibitz S, Miller JF, et al. Pertactin is required for Bordetella species to resist neutrophil-mediated clearance. Infect Immun. 2010;78(7):2901-9. doi: 10.1128/IAI.00188-10. PubMed PMID: 20421378; PubMed Central PMCID: PMCPMC2897405.
2. Posfai G, Kolisnychenko V, Bereczki Z, Blattner FR. Markerless gene replacement in Escherichia coli stimulated by a double-strand break in the chromosome. Nucleic Acids Res. 1999;27(22):4409-15. doi: 10.1093/nar/27.22.4409. PubMed PMID: 10536150; PubMed Central PMCID: PMCPMC148724.
3. Kovach ME, Phillips RW, Elzer PH, Roop RM, 2nd, Peterson KM. pBBR1MCS: a broad-host-range cloning vector. Biotechniques. 1994;16(5):800-2. PubMed PMID: 8068328.
4. Kovach ME, Elzer PH, Hill DS, Robertson GT, Farris MA, Roop RM, 2nd, et al. Four new derivatives of the broad-host-range cloning vector pBBR1MCS, carrying different antibiotic-resistance cassettes. Gene. 1995;166(1):175-6. doi: 10.1016/0378-1119(95)00584-1. PubMed PMID: 8529885.
