# Supplementary material for: Cytotoxicity of the effector protein BteA was attenuated in Bordetella pertussis by insertion of an alanine residue
Source: PLoS Pathog. 2020 Aug 10;16(8):e1008512. doi: 10.1371/journal.ppat.1008512 (PMC7446853; doi:10.1371/journal.ppat.1008512)
Supplement: S3 Table — The analyzed strain, primer sequence (5′-3′) and its position in chromosome are provided. (PDF) [file ppat.1008512.s004.pdf]

S3 Table. List of PCR primers used for verification of *Bordetella* chromosome mutations. The analyzed strain, primer sequence (5′-3′) and its position in chromosome are provided.

**Analyzed strain / mutation**	**Primer sequence for / rev**		**PCR position in chromosome**
*Bb*D445 Δ*bscN*	for<br>rev	GAATTTCGCCGTGTCTTCCG<br>GACAAGGTGGTGTCGGGTAG	3931750-3931769<br>3928676-3928695
*Bb*D445 Δ*bteA*	for<br>rev	CGTGCATTGCATCTACCCGT<br>AGGCCGTCAATCACCACATT	3179894-3179913<br>3183446-3183465
*Bb*D445 *bteA*insA503	for<br>rev	AGCAGGCCGACGACGACAG<br>CAACGATGGCGATATCGAATTGCGC	3181508-3181526<br>3182774-3182798
*Bp*B1917 Δ*bscN*	for<br>rev	GAATTTCGCCGTGTCTTCCG<br>GACAAGGTGGTGTCGGGTAG	2286186-2286205<br>2283112-2283131
*Bp*B1917 Δ*bteA*	for<br>rev	CGTGCATTGCATCTACCCGT<br>AGGCCGTCAATCACCACATT	3764969-3764988<br>3761421-3761440
*Bp*B1917 *bteA*ΔA503	for<br>rev	AGCAGGCCGACGACGACAG<br>CAACGATGGCGATATCGAATTGCGC	3763357-3763375<br>3762088-3762112
*Bp*B1917 Δ*btrA*	for<br>rev	CTTGGCGTCTATCCCGATCC<br>CCGACCATGAGGATCACGTC	2274843-2274862<br>2276678-2276697
