# Supplementary material for: Cytotoxicity of the effector protein BteA was attenuated in Bordetella pertussis by insertion of an alanine residue
Source: PLoS Pathog. 2020 Aug 10;16(8):e1008512. doi: 10.1371/journal.ppat.1008512 (PMC7446853; doi:10.1371/journal.ppat.1008512)
Supplement: S4 Table — Name, description and reference are indicated. (PDF) [file ppat.1008512.s005.pdf]

S4 Table. List of mammalian and yeast cells used in this study. Name, description and reference are indicated.

	**Genotype and relevant description**	**Reference**
Mammalian cells		
HeLa	Human cervical adenocarcinoma cell line	ATCC, CCL-2™
Saccharomyces cerevisiae strains		
BY4741	*MAT*a; *his3*Δ1; *leu2*Δ0; *ura3*Δ0; *met15*Δ0	Euroscarf, Germany
BY4741 / pYC2-CT	BY4741 yeast strain harboring mock pYC2-CT	this study
BY4741 / *Bb*RB50 *bteA-GFP*	BY4741 yeast strain harboring pYC2-CT-encoded *bteA* allele of *Bb*RB50 fused C-terminally with *GFP*	this study
BY4741 / *Bb*RB50 *bteA*insA503-*GFP*	BY4741 yeast strain harboring pYC2-CT-encoded mutant *Bb*RB50 *bteA*insA503 allele fused C-terminally with *GFP*	this study
BY4741 / *Bp bteA-GFP*	BY4741 yeast strain harboring pYC2-CT-encoded *bteA* allele of *Bp*B1917 fused C-terminally with *GFP*	this study
BY4741 / *Bp bteA*ΔA503-*GFP*	BY4741 yeast strain harboring pYC2-CT-encoded mutant *Bp*B1917 *bteA*ΔA503 allele fused C-terminally with *GFP*	this study
