# Supplementary material for: Cytotoxicity of the effector protein BteA was attenuated in Bordetella pertussis by insertion of an alanine residue
Source: PLoS Pathog. 2020 Aug 10;16(8):e1008512. doi: 10.1371/journal.ppat.1008512 (PMC7446853; doi:10.1371/journal.ppat.1008512)
Supplement: Attachment — Submitted filename: Letter Revision Bayram et al.docx [file ppat.1008512.s006.docx]

Dear Editor,

Thank you for your letter of May 22, inviting us to resubmit a revised version of the manuscript PPATHOGENS-D-20-00592 by Bayram *et al.*, entitled: “Cytotoxicity of the effector protein BteA was attenuated in *Bordetella* *pertussis* by insertion of an alanine residue”.

We wish to thank the reviewers for critical reading and thoughtful comments that helped us to improve the manuscript. The suggestions were duly considered and followed. The data shown in Fig. S1 was now incorporated into the modified Fig. 3A and an additional LD50 experiment was performed in order to enable proper statistical evaluation of the data, as suggested by reviewer #2. The wording suggestions of the reviewers were also reflected in the revised text.

Please, find attached a detailed point-by-point response to the initial reviews and the list of the modifications performed in the revised manuscript.

Hoping that the revised manuscript will now meet the standards of PLOS Pathogens, I wish to thank you for your editorial work.

Sincerely yours,

Jana Kamanova

(on behalf of all co-authors)

Response to reviewers of the manuscript PPATHOGENS-D-20-00592:

*Reviewer #1:*

***Issue:*** *Although the finding are important to give an idea for the cause of differences between the activity of the two homologs, the authors lack a good explanation for this and do not give a mechanism for this changes. I do miss some secondary structure analysis that may indicate for the position of this insertion and if it is exposed or located in a place that might do something.*

**A:** The reviewer raised a difficult issue that has been a source of quite some frustration over the past years. Unfortunately, the structure of BteA and the mechanism of its cytotoxic action, as well as the biochemistry behind, remain unknown. We have been trying to build structural models of BteA, including *ab initio* modeling, but in the absence of any solved structure of any homologous protein, these models have proven quite speculative and useless. Insertion of charged or aromatic residues instead of Ala at position 503 had the same impact on cytotoxic activity of BteA (data not shown), suggesting that the side chain of the inserted residue does not play a role and the disruptive effect of A503 insertion comes from an alteration of the backbone structure. However, the secondary structure predictions would suggest that the A503 residue might be located within an unstructured region of the protein, and the formulation of any useful hypothesis was not possible.

Reviewer #2:

***Issue 1:*** *Although the A503 insertion is highly attenuating, residual activity can be detected as shown in Fig. S1. The authors should elevate this histogram to become Fig. 1E. It shows a comparatively low level of cytotoxic activity for Bp BteA when the T3SS is derepressed. The residual cytotoxic activity could simply be that, or it could be due to some other activity of BteA that only indirectly affects viability. Regardless, the function, if any, of the most commonly circulating B. pertussis BteA allele seems important to thoroughly understand.*

**A1:** The suggestion of the reviewer was followed and the data from the histogram of Fig S1 was now incorporated into Fig. 3A, since Fig. 1 deals with cytotoxicity provoked by *B. bronchiseptica* strains secreting BteA variants. The new Fig. 3A now allows a direct comparison of the time course of cytotoxicity at 3, 6 and 24 h post-infection that results from deregulated production of *Bp* BteA in the Δ*btrA* mutant of *B. pertussis* and the increase of specific cytotoxic activity of the *Bp* BteA effector due to removal of the A503 residue. We have also modified Fig. 3B accordingly to match the strains analyzed in Fig. 3A.

***Issue #2:*** *The Discussion could be tightened up in a few places and would benefit from some pruning. In addition, clearly pointing out the discrepancy between the data presented here and the French et al. 2009 paper is important, but I suggest toning it down a bit. There are many factors that might account for such differences, and the data presented in this manuscript are clear and convincing.*

**A2:** The suggestion was followed, and the Discussion was streamlined and handling of the discrepancy between data presented here and data of French *et al.* 2009 was duly toned down.

***Issue #3:*** *The survival plots in Fig. 3C and 3D need appropriate statistics. All I could find is a general statement that the results were reproduced in 2 experiments using 6 mice per challenged group and challenge dose.*

**A3**: The recommendation was followed. A third LD50 experiment was performed to allow proper statistical evaluation of the data and determination of the *p*-value of the calculated LD50 values for the wild type and *bteA*ΔA503 mutant strains (n=3) using unpaired two-tailed *t*-test (*p* < 0.01, *Bp* WT vs. *Bp bteA* ΔA503). The new survival plots shown in Fig. 3C now also compare the survival curves of the *bteA*ΔA503 and of the parental *B. pertussis* strains at the same inoculation doses, which allowed survival distribution analysis using the long-rank test (*p* < 0.001 at medium and high inoculation dose, *Bp* WT vs. *Bp bteA* ΔA503). The statistical treatment is now outlined in the revised manuscript.
